# Biochemistry and Nanomechanical Properties of Human Colon Cells upon Simvastatin, Lovastatin, and Mevastatin Supplementations: Raman Imaging and AFM Studies

**DOI:** 10.1021/acs.jpcb.2c03724

**Published:** 2022-09-09

**Authors:** Karolina Beton, Beata Brożek-Płuska

**Affiliations:** Laboratory of Laser Molecular Spectroscopy, Institute of Applied Radiation Chemistry, Lodz University of Technology, Wroblewskiego 15, 93-590 Lodz, Poland

## Abstract

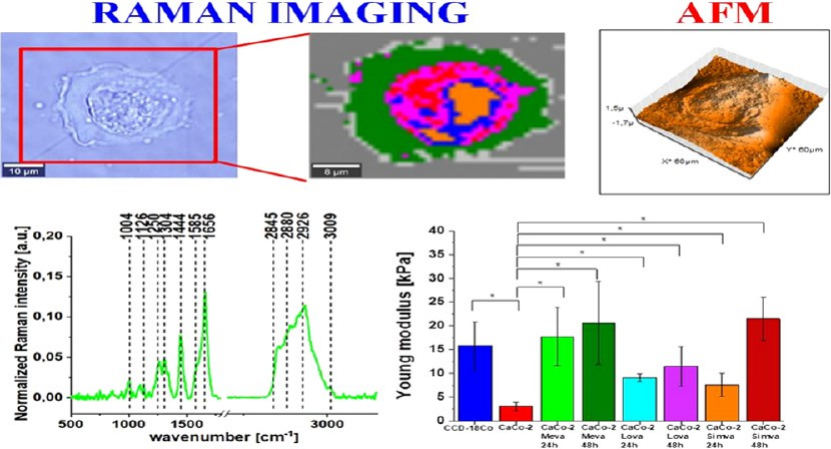

One of the most important areas of medical science is oncology, which is responsible for both the diagnostics and treatment of cancer diseases. Over the years, there has been an intensive development of cancer diagnostics and treatment. This paper shows the comparison of normal (CCD-18Co) and cancerous (CaCo-2) cell lines of the human gastrointestinal tract on the basis of nanomechanical and biochemical properties to obtain information on cancer biomarkers useful in oncological diagnostics. The research techniques used were Raman spectroscopy and imaging and atomic force microscopy (AFM). In addition, the studies also included the effect of the statin compounds—mevastatin, lovastatin, and simvastatin—and their influence on biochemical and nanomechanical changes of cell properties using Raman imaging and AFM techniques. The cytotoxicity of statins was determined using XTT tests.

## Introduction

Cancer development is a complex multistage process related to the transformations of normal cells to pathological ones. Colorectal cancer (CRC) is the second most common cancer in both men and women worldwide and is the leading cause of death. The mortality rate related to this type of cancer is high and approximately equal to 60% in Europe and the USA.^1^ Moreover, CRC is characterized by high metastasis.^2−4^ The risk factors for CRC can be divided into three main groups: (1) environmental (e.g., high-fat diet, high-calorie diet, and diet low in silage, vegetables, and fruit), (2) internal (e.g., adenomas, ulcers, Crohn’s syndrome), and (3) genetic (e.g., familial adenomatous polyposis).^5^ Around 75–95% of CRC cases occur in people without any genetic load, which makes lifestyle and eating habits particularly important in this type of cancer development.^6,7^

**Figure 1:**
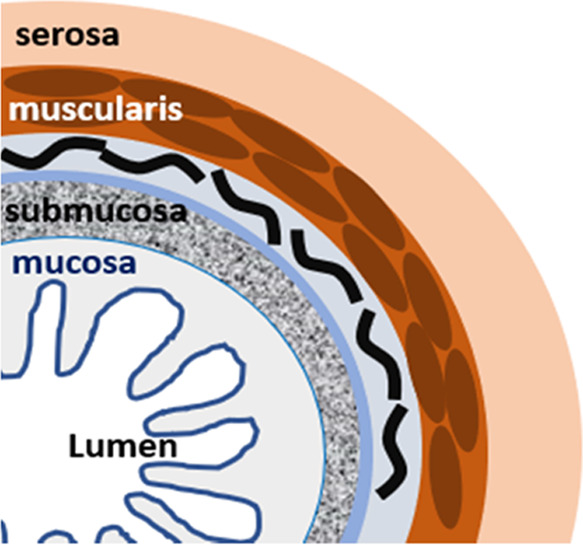
Cross section through the layers of the human colon.

Generally, in the first stage of CRC development, healthy cells in the lining of the colon change, grow, and divide uncontrollably to form a mass of tumor. Both genetic and environmental factors can change the dynamics of this process. CRC most often begins with a polyp, a noncancerous growth that can develop on the inner wall of the colon and then can transform into cancer or metastatic cancer. Figure 1 presents the cross section through the layers of the human colon.

The first *in vivo* Raman measurements of human gastrointestinal tissue were published in 2000 by Shim et al.^8^ This study showed that Raman spectroscopy can be successfully used for disease classification during *in vivo* examination using fiber probes coupled with a Raman spectrometer. Raman studies of CRC have been published also by Andrade et al.^9^ The authors developed the diagnostic algorithm useful to establish the spectral differences of the complex colon tissues to find the characteristic Raman features of cancerogenesis. Popp’s group performed the first CARS measurements of colon tissue samples.^10^ The comparison of the CARS results with those obtained using the typical Stokes component of Raman spectra showed many similarities, simultaneously underlying the main advantage of the CARS technique—the short acquisition time. Liu et al. used chemometric methods: principal component analysis (PCA) and partial least-squares-discriminant analysis (PLSDA) to prove that Raman spectra can be effectively used to differentiate normal and cancerous human colon tissues.^11^ In 2006, single living cells of the epithelium of CRC and control mucosa were analyzed by Raman spectroscopy by Chen et al.^12^ PCA revealed a separation between epithelial cells of mucosa and cancerous tissues according to spectral signals assigned to nuclei and proteins with the sensitivity of 77.5% and specificity of 81.3%.^12^

Nowadays, early-stage cancer detection and margin detection of cancerous lesions are still challenging. The diagnostics is often based on invasive techniques, whether a suspicious change—for example, a colon polyp must be removed from the patient’s body. Depending on the organ and the location of the suspicious tissue area, different biopsy methods can be used: punching out a cylinder of tissue (punch biopsy), aspiration of tissue or cells (fine-needle biopsy, fine-needle puncture), or sampling of tissue with a scalpel (excisional biopsy) or endoscopically with tiny forceps. It must be highlighted that all of these procedures are time-consuming and expensive.

Also, accurately detecting cancer is a crucial and foremost step toward improving the survival rate of patients with colorectal cancer. Currently, colonoscopy and histopathology are standard screening and diagnostic techniques for colorectal tissues. Although colonoscopic screening has significantly increased the survival rate of patients with colorectal cancer, it remains a challenge to distinguish adenomas and early adenocarcinomas from benign hyperplasticpolyps using colonoscopy. Immunohistochemistry also has limitations due to the difficulty of analyzing large volumes of tissue sections by staining and the inability to detect multiple signals simultaneously. Also, the reproducibility and robustness of genomic data remain a concern due to the heterogeneity of tumors. There is, therefore, a real need to develop robust diagnostic and classification tools that have reproducibility and translational application with clinical samples.

Several studies have shown that spectral histopathology (SHP) is capable of classifying different tissue types and especially cancer cells.^13−18^ The advantage of spectroscopic techniques correlates with the fact that the measured vibrational spectra are integral signals of the proteome, genome, and metabolome. In other words, in one measurement, it is possible to obtain complex information about the sample and determine, e.g., the health or cancer status. Thus, when vibrational spectra are collected from distinct regions of tissue sections, variations in the spectral patterns can be detected and can be correlated with cancer areas.^13−18^

Raman microspectroscopy and imaging provide label-free identification and localization of cancer based on many signals. Biomolecules such as proteins, lipids, or nucleic acids are Raman-active and thus provide molecular fingerprints that are highly sensitive and can reflect a specific tissue state or cellular phenotype. That is why the development of new, spectroscopic cancer diagnostic methods in the form of SHP is extremely valuable.

**Figure 2:**
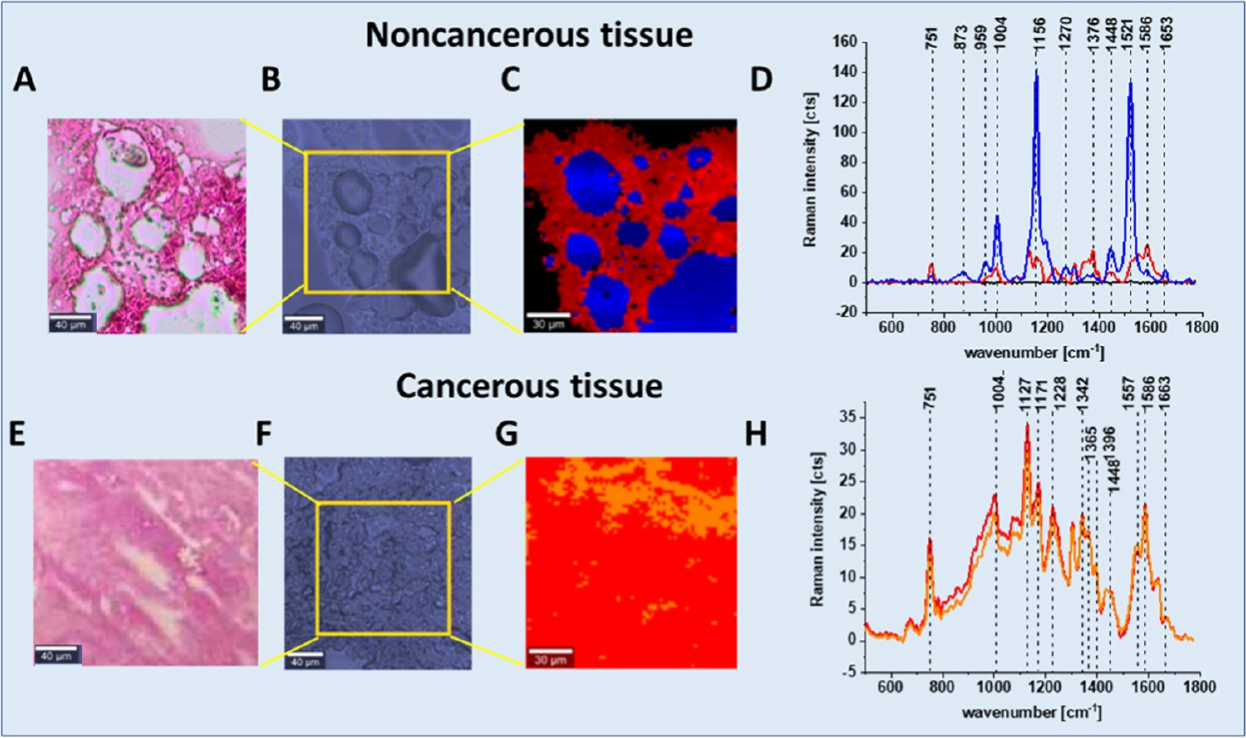
Histological image (A, E), microscopy image (B, F), Raman image (C, G), and the average Raman spectra (D, H) typical for noncancerous and cancerous human colon tissues. The white bar in the pictures, on the lower left corner, is the inner scale, with values of 40 μm (A, B, E, F) and 30 μm (C, G).

Figure 2 shows the histological image (A, E), microscopy image (B, F), Raman image (C, G), and the average Raman spectra (D, H) typical for noncancerous and cancerous human colon tissues.

The other current challenge is cancer treatment. Some promising anticancer drugs are statins. Statins are understood to indicate a group of organic, multifunctional chemical compounds. They occur both naturally and are made synthetically in laboratories. Depending on the structure of the statin compound mevastatin, lovastatin, pravastatin, compactin (statins of natural origin), simvastatin (semisynthetic statins), atorvastatin, rosuvastatin, pitavastatin, cerivastatin, and fluvastatin (synthetic statins) can be distinguished. All of these compounds have a pharmacophore group in their active form. Due to their pleiotropic effects, statins are also being used in oncology.^19^ In experiments, both *in vitro* and *in vivo*, statins inhibited the proliferation of cancer cells, induced apoptosis, i.e., programmed cell death, and reduced the number of metastases or delayed their occurrence.^20^ They have also been observed to work synergistically with many drugs, not only with standard chemotherapeutics (cisplatin, doxorubicin) but also with preparations not used in cancer treatment (bisphosphonates, saquinavir).^21−24^

Generally, statins inhibit 3-hydroxy-3-methyl-glutaryl-coenzyme A (HMG-CoA) reductase, the enzyme that converts HMG-CoA into mevalonic acid (MVA), a cholesterol precursor, but statins do more than just compete with the normal substrate in the enzymes active site. They alter the conformation of the enzyme when they bind to its active site. This prevents the HMG-CoA reductase from attaining a functional structure. The change in conformation at the active site makes these drugs very effective and specific. Moreover, binding of statins to HMG-CoA reductase is reversible.^25^ The inhibition of HMG-CoA reductase determines the reduction of intracellular cholesterol, inducing the activation of a protease, which slices the sterol regulatory element binding proteins (SREBPs) from the endoplasmic reticulum. SREBPs are translocated at the level of the nucleus, where they increase the gene expression for the LDL receptor. The reduction of cholesterol leads to the increase of LDL receptors, which determines the reduction of circulating LDL and of its precursors (intermediate density lipoprotein (IDL) and very low-density lipoprotein (VLDL)).^26^ All statins reduce LDL cholesterol nonlinearly, dose-dependently, and after administration of a single daily dose.

At least four mechanisms were proposed to explain statins’ antioxidant properties. (1) The hypocholesterolemic effect, resulting in reduced lipoprotein cholesterol, and thus, a reduced level of oxidation substrate. (2) The decrease in cell oxygen production, by inhibiting the generation of superoxide by macrophages. Recently, it was demonstrated that statins can attenuate the formation of the superoxide anion in endothelial cells by preventing the prenylation of the p21 Rac protein.^27^ Statins can also prevent LDL oxidation by preserving the activity of the endogenous antioxidant system, like superoxide dismutase.^28^ (3) The binding of statins to phospholipids on the surface of lipoproteins preventing diffusion toward the lipoprotein core of free radicals generated during oxidative stress. (4) The potent antioxidative potential of the metabolites also results in lipoprotein protection from oxidation.

Because statins are structural analogues of 3-hydroxy-3-methyl-glutaryl-coenzyme A (HMG-CoA), they compete with it for the active site of HMG-CoAR. As statins bind to the enzyme more strongly than its natural substrate, the reduction of HMG-CoA and the production of mevalonic acid (MVA) are inhibited.^29,30^ Due to the fact that the cellular concentration of MVA depends on the activity of HMG-CoAR, and MVA is necessary for the subsequent reactions of the cholesterol synthesis pathway, this step is considered crucial for the whole process. For this reason, statins are used in the treatment of hypercholesterolemia.^29−34^ Moreover, statins increase the number of receptors for low-density lipoproteins on the surface of hepatocytes, which increases the absorption of cholesterol and additionally reduces its concentration in the blood.^30,33,35,36^ Statins inhibit the progression of atherosclerosis and reduce the number of cardiovascular events in patients with ischemic heart disease (IHD).^37−39^ The beneficial effects of statin use in the treatment of IHD were also noted in patients with normal cholesterol levels, which suggests that statins also act in a mechanism independent of their cholesterol-lowering effect.^40^ Indeed, statins act on the cell and the body through several independent mechanisms. Due to their pleiotropic effect, the positive effects of their use are observed in the treatment of many diseases.^41^ Statins have antiplatelet,^42^ antihypertensive,^43,44^ and anti-inflammatory properties.^45,46^ Since the main indication for the use of statins is lipid disorders, which are a common disease, and this group of drugs is also used in other diseases, statins are among the most commonly prescribed drugs. Currently, there are reasons to use them also in the case of cancer.

The fact that mevalonate plays a key role in cell proliferation and that many malignant cells present an increased HMG-CoA reductase activity suggest that selective inhibition of this enzyme could lead also to new chemotherapy for cancer disease. The obtained reduction of sterol synthesis by statins suggests that inhibition of tumor cell growth can be related to the reduction of nonsteroidal isoprenoid compounds. The inhibitory effect on the synthesis of isoprenoid compounds formed in the side branches of the MVA pathway may play an important role in the anticancer properties of statins. These substances include dolichol, ubiquinone, isopentenes-loadenosine, geranylgeranyl pyrophosphate (GGPP), or farnesyl pyrophosphate (FPP).^47^ Dolichol phosphate is also a carrier of extracellular sugar residues of proteoglycans, the effect of which can be associated with gene expression and with the change in antigenic properties of the cell membrane, intercellular interactions, and the flow of information in signaling pathways.^48^ The reduction in mevalonate synthesis also leads to a reduction in intracellular concentrations of farnesyl pyrophosphate (FPP) and geranylgeranyl (GGPP). These proteins are responsible for the growth, differentiation, apoptosis, and modulation of the actin cytoskeleton of cells and thus for cell migration and adhesion. Mutant Ras or Rho proteins are typical for cancers.^49^

In the presented studies, the innovative combination of biochemical and nanomechanical characterization of human colon cells—normal CCD-18Co, cancer CaCo-2, and cancer CaCo-2—upon statin supplementation using Raman spectroscopy and imaging and atomic force microscopy (AFM) techniques will be presented. The influence of statin type will also be discussed.

## Materials and Methods

### Cell Lines and Cell Culture

The research subjects were CCD-18Co (ATCC CRL-1459) and Caco-2 (ATCC HTB-37) cell lines purchased from ATCC: The Global Bioresource Center. The CCD-18Co cell line was cultured in accordance with the manufacturer’s recommendations using ATCC-formulated Eagle’s minimum essential medium with l-glutamine (catalog No. 30-2003). To make the complete growth medium, fetal bovine serum was added to a final concentration of 10%. The complete culture medium was renewed every 2–3 days. The cells CCD-18Co were obtained from a patient, and their characteristics and morphology are normal myofibroblasts in the colon. The CaCo-2 cell line was also cultured according to the ATCC protocols. The CaCo-2 cell line was obtained from a patient—a 72-year-old Caucasian male diagnosed with colon adenocarcinoma. To make the medium complete, we based it on Eagle’s minimum essential medium with l-glutamine (catalog No. 30-2003), with the addition of a fetal bovine serum to a final concentration of 20%. The medium was renewed once or twice a week.

The biological safety of both CCD-18Co and Caco-2 cell lines has been classified by the American Biosafety Association (ABSA) as level 1 (BSL-1).

### Cultivation Conditions

Cell lines (CCD-18Co, Caco-2) embraced in the experiments in this study were grown in flat-bottom culture flasks made of plasma-treated polystyrene with a cell growth surface of 75 cm^2^. Flasks containing cells were stored in an incubator providing environmental conditions at 37 °C, 5% CO_2_, and 95% air.

### Raman Spectroscopy and Imaging

**Figure 3:**
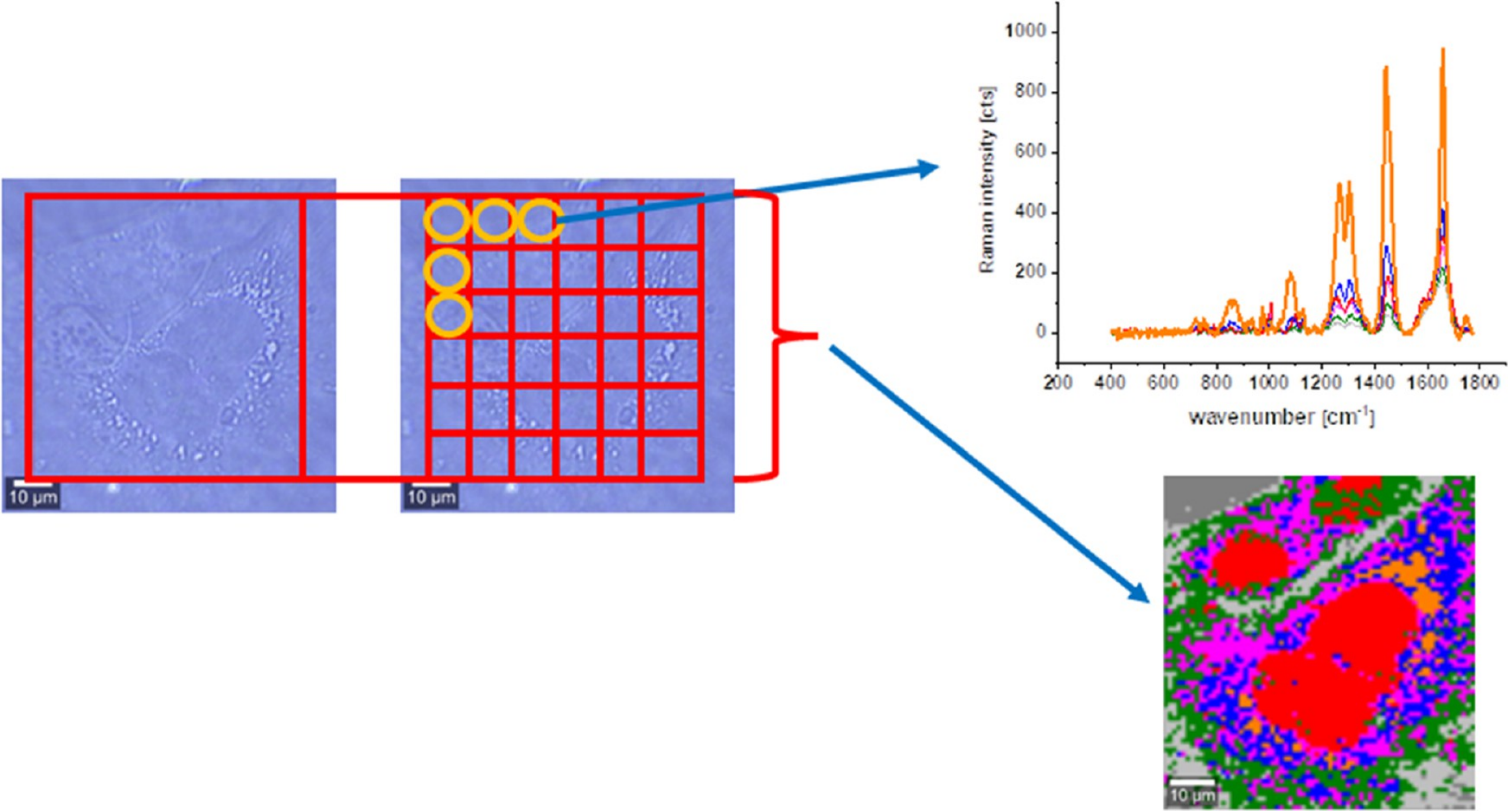
Schematic comparison of Raman single spectra and Raman imaging modes of data acquisition. The scale bar is the same for all images and is equal to 10 μm.

All Raman spectra and images presented and discussed in this paper were registered using the confocal microscope Alpha 300 RSA+ (WITec, Ulm, Germany) equipped with an Olympus microscope integrated with an optical fiber with a 50 μm core diameter coupled with an ultrahigh throughput spectrometer (UHTS) and a charge-coupled device (CCD) camera (Andor Newton DU970NUVB-353) operating in the default mode at −60 °C in full vertical binning. Laser with an excitation line 532 nm was focused on the sample through a Nikon objective lens with a magnification of 40× and a numerical aperture (NA = 1.0) intended for cell measurements performed by immersion in phosphate-buffered saline (PBS). The average excitation power of the laser during the experiments was 10 mW, with an integration time of 0.5 s for Raman measurements for the high-frequency region and 1.0 s for the low-frequency region. An edge filter was used to filter out the Rayleigh scattered light, which means that the reflected light that reaches the detector comes only from the plane from which the image is created. A piezoelectric table was applied to set the sample in the right place by manipulating the *XYZ* positions and consequently record Raman images. Spectra were collected with one acquisition per pixel and a diffraction grating of 1200 lines/mm. Cosmic rays were removed from each Raman spectrum (model: filter size: 2; dynamic factor: 10), and the Savitzky–Golay method was implemented for the smoothing procedure (order: 4; derivative: 0). All data were collected and processed using a special original software WITec Project Plus. Figure 3 shows the comparison of Raman single spectra and Raman imaging modes of data acquisition.

All imaging data were analyzed by cluster analysis (CA), which allows for grouping of a batch of vibrational spectra that bear similarity to each other. CA was accomplished using WITec Project Plus software with the Centroid model and *k*-means algorithm, in which each cluster is represented by one vector of the mean. The normalization, model: divided by norm (divide the spectrum by the data-set norm), was performed using Origin software according to the formula
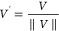
1
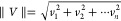
2where *ν*_*n*_ represents the *n*th *V* values.

The normalization was performed for all Raman spectra presented in the manuscript.

The Origin software was also used to perform analysis of variance (ANOVA) necessary to indicate statistically significant results (means comparison: Tukey’s model; significance level: 0.05).

### AFM Measurements

AFM measurements were performed using a PIK Instruments atomic force microscope in the scanning range of 100 × 100 μm^2^ in the *X* and *Y* axes and at 15 μm in the *Z* axis with a positioning resolution in the *XY* axis of 6 pm and in the *Z* axis of 0.9 pm, equipped with an inverted microscope, enabling measurements in air and liquid, in both contact and tapping modes. Nanosurf C3000 software was used for AFM data collection. During measurements, topography maps and nanomechanical properties of cells with and without supplementation of statins were determined with the resolution of 256 × 256 points per 60 × 60 μm^2^. qp-Bio-AC-50 tips produced by nanosensors with a spring constant of 0.6 N/m were used. The analysis of AFM data was performed using AtomicJ software^50^ to obtain information about Young’s modulus of analyzed biological samples.

For AFM measurements, cells were cultured on Petri dishes filled with a culture medium. Once the growing cells formed a semiconfluent monolayer, the dish with cells was mounted on the AFM scanner, the medium was replaced by PBS, and the sample was measured within the next 2–3 h (at room temperature and ambient conditions).

### Chemical Compounds

Mevastatin (M2537-5MG), simvastatin (S6196-5MG), and lovastatin (PHR1285-1G) were purchased from Sigma-Aldrich and used without additional purification. The XTT proliferation kit with catalogue Number 20-300-1000 was purchased from Biological Industries.

### XTT

One application of the XTT colorimetric assay is to test the viability of cells as a function of the compound that is active on them and the concentration of the compound. An example of this type of compound is statin. In the publication by Ludwig et al., the effect of three statins was investigated: atorvastatin, simvastatin, and pravastatin.^51^ For this purpose, a test was performed for each of the compounds for different concentrations of the tested substance. The statin compound was added after placing normal endothelial or cancer cells (CPAEs) in a 96-well plate medium and after incubating the cells for 24 or 48 h. In addition, a control was performed with only cells submerged in the pure culture medium. Then, after the addition of statins, the cells were incubated again for 3 h with formazan salts, after which it was possible to perform the measurement. Based on the obtained results, the survival curves of the studied cells were determined depending on the statin compound used.

### Determination of the Appropriate Statin Concentration Using the XTT Test

For each cell type, XTT tests were performed 24 and 48 h after the addition of mevastatin, lovastatin, and simvastatin to the cells immersed in the culture medium. Preparation for the test included proper filling of the 96-well plate according to the procedure developed at the Institute of Applied Radiation Chemistry in Lodz. The wells were filled in such a way that each row contained a specific series of measurements. For example, in one row, all plates were filled with a medium; in another, control samples containing only cells were immersed in the medium; and only in subsequent rows, there were cells in the medium with the addition of a specific concentration of the selected statin. Six different concentrations of each statin (mevastatin, lovastatin, simvastatin) were selected for the test: 1, 5, 10, 25, 50, and 100 μM. After completing each of the 96-well plates, the samples were incubated at 37 °C for 24 or 48 h. After the time from the addition of statin, the XTT compound was added and the test was performed using the BioTek Synergy HT apparatus. The experiment was carried out after 3 h from the addition of the reagent containing formazan salts. After the completion of the study, the obtained results had to be analyzed using a spreadsheet, resulting in a bar graph showing the effect of added statin concentration on the survival of the tested cell type, taking into account the time since the addition of each statin.

In our previous paper in which we investigated cancer human colon cells (CaCo-2), it was found that for cells, the most appropriate concentration of mevastatin in the solution with a medium would be 10 μM.^52^ For each test, cell survivability at such a concentration fluctuated in the range of 50–60%, which made it possible to conclude that at such a concentration, the effect of mevastatin on cells will be noticeable in the study of both nanomechanical and biochemical properties, and there will be enough living cells to allow conducting of analyses.

**Figure 4:**
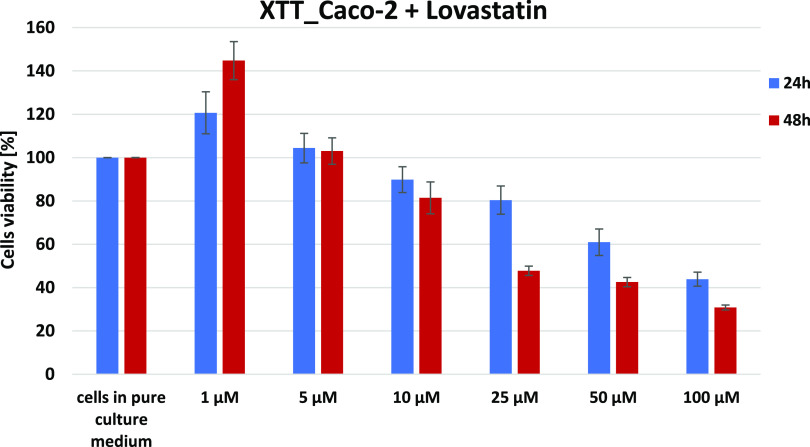
Results of the XTT comparison of the percent viability for Caco-2 human colon cancer cells supplemented with different concentrations of lovastatin in two different time intervals with the standard deviation ±SD.

Figure 4 shows the results of the XTT test obtained for Caco-2 human colon cancer cells supplemented with lovastatin in various concentrations and in different time intervals.

As indicated by the obtained results, the most optimal concentration for the observation of the effect of lovastatin supplementation is, similar to the previous type of statin, 10 μM. Moreover, the concentration of the maintenance of a constant statin concentration of 10 μM enables reference to the comparison of the effect of other statins and to the previously published work describing the effect of mevastatin, as we mentioned above.^52^

To summarize, taking into account the previously published results for mevastatin, and presented data for lovastatin, we decided to use the same concentration for the other types of statins to compare their effect and compare the results to those obtained previously. The experimental idea applied in this way allows the most precise and unambiguous way to determine the effect of individual statins of the same concentration on the basic functions of the cell and to track possible biochemical changes.

In the presented studies, in the further part of the experiments (Raman spectroscopy and imaging and AFM), the effect of statins only on colon cancer cells (CaCo-2) after 24 and 48 h was investigated because it is well known from the literature that statins are not destructive in normal cells, as is the case with most drugs used to treat cancer.^21,29,53−56^

It is known from the literature that lovastatin increases the concentration of cyclin inhibitors in the cell, arresting the G1 phase of the cancer cell cycle.^57^ Due to the ability of statins to block proteasomal degradation of proteins,^58,59^ they show activity independent of the MVA pathway. The inhibitory effect of the proteasome, however, becomes apparent only at relatively high doses of statins. The activation of the peroxisome proliferator-activated receptor-g has been recently described as an additional antitumor mechanism of action for statins. It induces the production of the tumor-suppressor gene, which is accompanied by a decrease in the phosphorylation of protein kinase B and mitogen-activated protein kinases and the blockage of the cell cycle in the G1 phase.^60^

The role in the cytostatic effect of statins can also be attributed to the induction of differentiation in cancer cells. In conclusion, the antiproliferative effect of statins has been confirmed in the treatment of gastric, pancreatic, breast, lung cancer, colon adenocarcinoma, and acute myeloid leukemia.^61−64^ Normal cells are also subject to this action. Statins inhibit the growth of normal endothelial cells, smooth muscles, and fibroblasts.^65,66^ However, the effect of statins on normal cells is much weaker, probably due to a lower proliferative potential and greater demand for its products in cancerous cells.^47,67−69^ The cytostatic effect of individual statins on different tumor cell lines is not identical. The effect of their use depends primarily on the dose and chemical properties, as well as the type of tumor.

## Results

One of the main goals of our study was to determine the statistically significant differences between normal and cancer human colon cells, including cancer cells supplemented by mevastatin, simvastatin, and lovastatin based on their vibrational features. Therefore, to properly address these tasks, we investigated systematically how the Raman imaging and Raman spectroscopy methods respond to *in vitro* normal and cancer human cells without and upon the supplementation by statins.

Herein, we present a valuable, fast, and costless method for cell structure visualization and cells’ virtual staining, which adds the biochemical information given by the Raman intensity to the pseudo-color images. These label-free images with high spatial resolution enable a direct analysis of all human colon cell substructures, which can help track the biochemistry changes typical for cancerogenesis and can help in the analysis of anticancer treatment.

**Figure 5:**
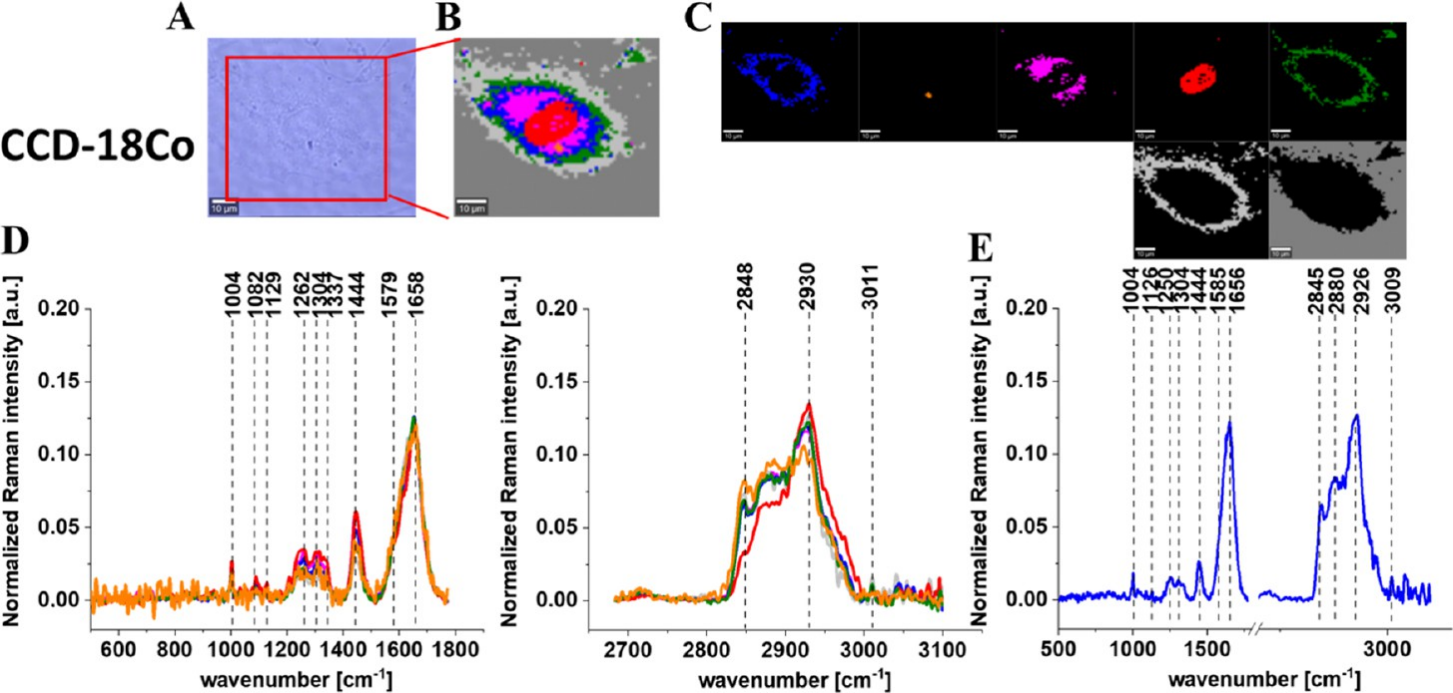
Microscopy image (A) and Raman image (B) constructed based on the cluster analysis (CA) method; Raman images of all clusters identified by CA assigned to lipid-rich regions (blue and orange), mitochondria (magenta), nucleus (red), cytoplasm (green), cell membrane (light gray), and cell environment (dark gray) (C); the average Raman spectra typical for all identified clusters for low-frequency and high-frequency regions (D); and the average Raman spectrum for the cell as a whole (E) for human normal colon cells CCD-18Co. All cells were measured in PBS. Colors of the spectra correspond to the colors of clusters, the excitation laser line was 532 nm, and the average Raman spectra were calculated based on the data for six cells. Adapted with permissions from ref (52). Copyright 2022 [Spectrochim. Acta, Part A].

**Figure 6:**
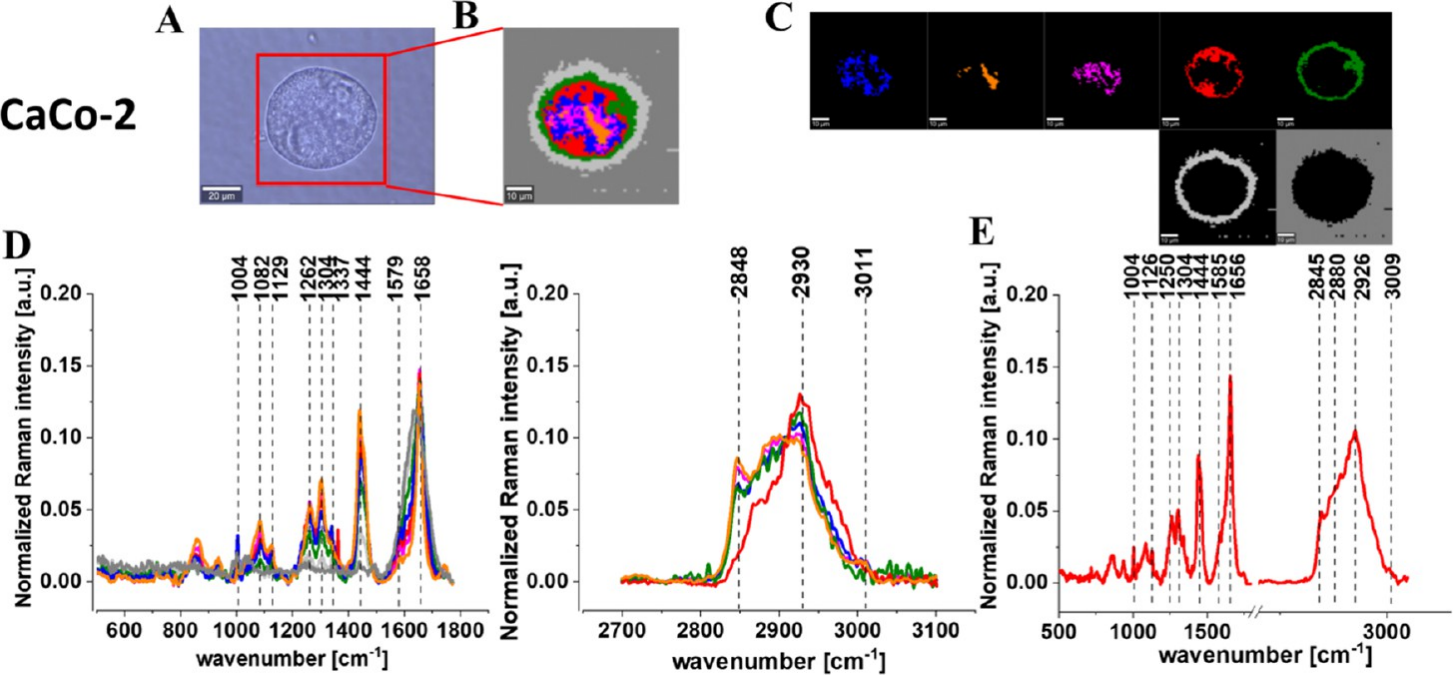
Microscopy image (A) and Raman image (B) constructed based on the cluster analysis (CA) method; Raman images of all clusters identified by CA assigned to lipid-rich regions (blue and orange), mitochondria (magenta), nucleus (red), cytoplasm (green), cell membrane (light gray), and cell environment (dark gray) (C); the average Raman spectra typical for all identified clusters for the low-frequency and high-frequency regions (D); and the average Raman spectrum for the cell as a whole (E) for human cancer colon cells CaCo-2. All cells were measured in PBS. Colors of the spectra correspond to the colors of clusters, the excitation laser line was 532 nm, and the average Raman spectra were calculated based on the data for six cells. Adapted with permission from ref (70). Copyright 2020 [Molecules].

**Figure 7:**
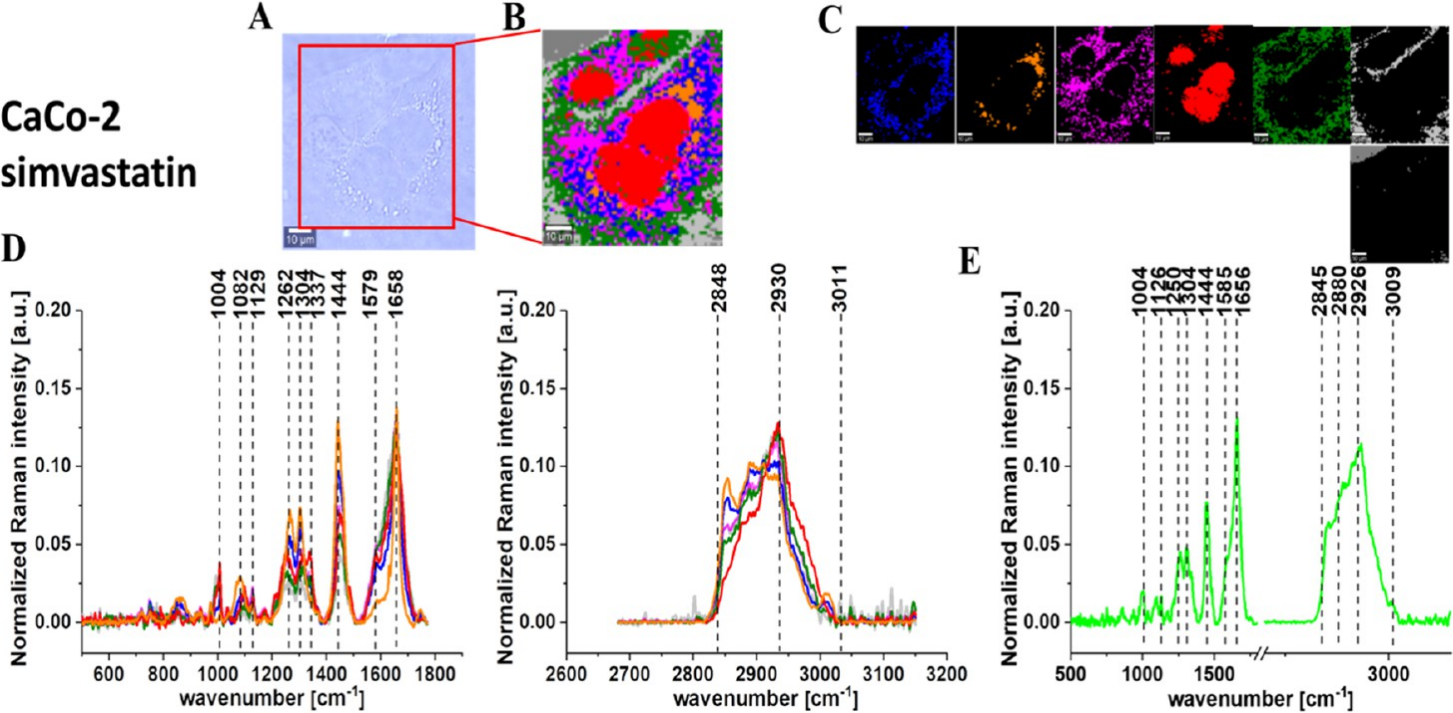
Microscopy image (A) and Raman image (B) constructed based on the cluster analysis (CA) method; Raman images of all clusters identified by CA assigned to lipid-rich regions (blue and orange), mitochondria (magenta), nucleus (red), cytoplasm (green), cell membrane (light gray), and cell environment (dark gray) (C); the average Raman spectra typical for all identified clusters for low-frequency and high-frequency regions (D); and the average Raman spectrum for the cell as a whole (E) for human cancer colon cells CaCo-2 upon supplementation with simvastatin in 10 μM concentration for 24 h. All cells were measured in PBS. Colors of the spectra correspond to the colors of clusters, the excitation laser line was 532 nm, and the average Raman spectra were calculated based on the data for six cells. The scale bar is the same for all images and is equal to 10 μm.

**Figure 8:**
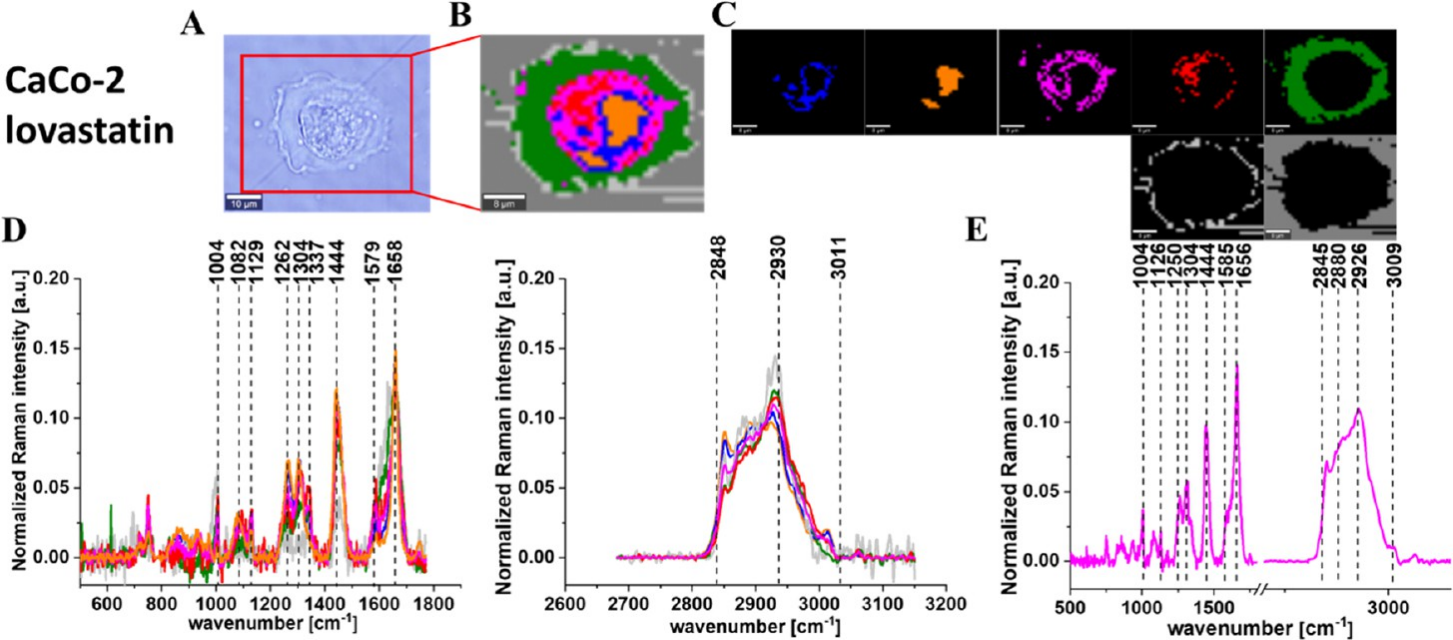
Microscopy image (A) and Raman image (B) constructed based on the cluster analysis (CA) method; Raman images of all clusters identified by CA assigned to lipid-rich regions (blue and orange), mitochondria (magenta), nucleus (red), cytoplasm (green), cell membrane (light gray), and cell environment (dark gray) (C); the average Raman spectra typical for all identified clusters for low-frequency and high-frequency regions (D); and the average Raman spectrum for the cell as a whole (E) for human cancer colon cells CaCo-2 upon supplementation with lovastatin in 10 μM concentration for 24 h. All cells were measured in PBS. Colors of the spectra correspond to the colors of clusters, the excitation laser line was 532 nm, and the average Raman spectra were calculated based on the data for six cells. The scale bar is the same for all images and is equal to 10 μm.

**Figure 9:**
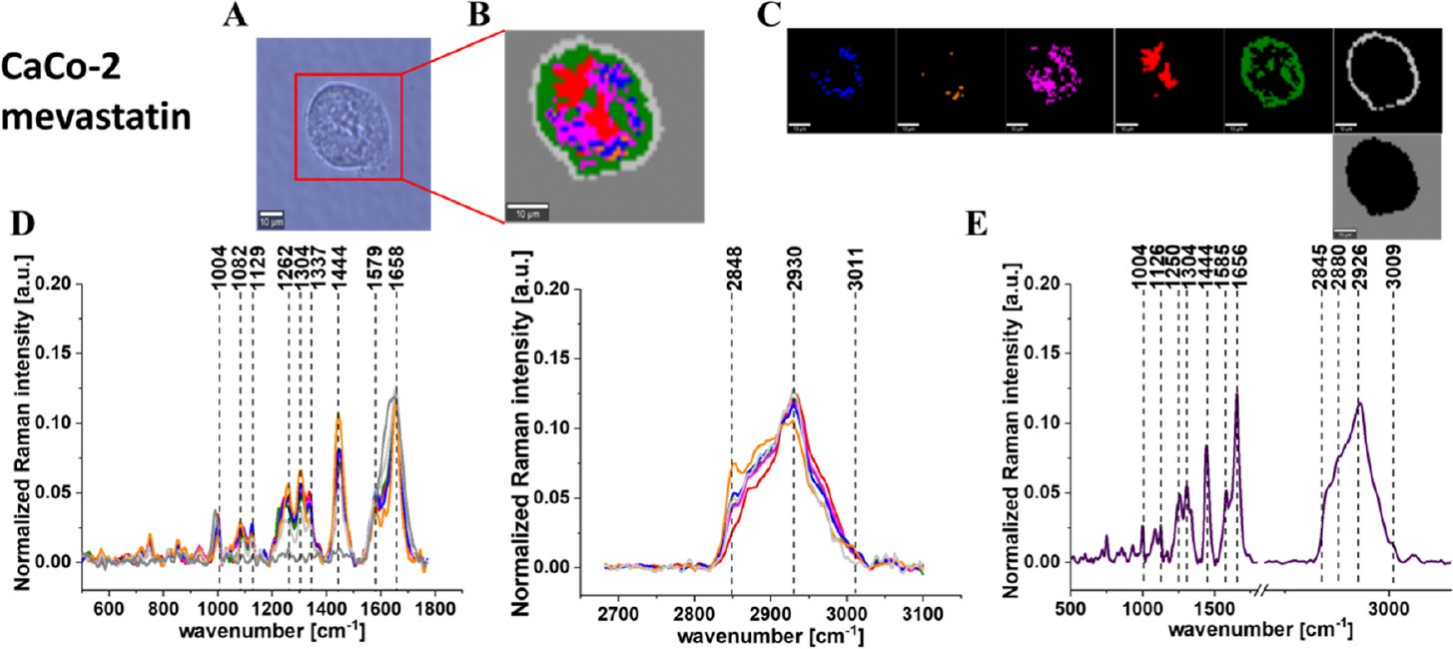
Microscopy image (A) and Raman image (B) constructed based on the cluster analysis (CA) method; Raman images of all clusters identified by CA assigned to lipid-rich regions (blue and orange), mitochondria (magenta), nucleus (red), cytoplasm (green), cell membrane (light gray), and cell environment (dark gray) (C); the average Raman spectra typical for all identified clusters for low-frequency and high-frequency regions (D); and the average Raman spectrum for the cell as a whole (E) for human cancer colon cells CaCo-2 upon supplementation with mevastatin in 10 μM concentration for 24 h. All cells were measured in PBS, Colors of the spectra correspond to the colors of clusters, the excitation laser line was 532 nm, and the average Raman spectra were calculated based on the data for six cells. The scale bar is the same for all images and is equal to 10 μm.

Figures 5–9 show the microscopy image, Raman image, and Raman images of all cell substructures identified using the cluster analysis algorithm; the average Raman spectra typical for identified lipid-rich structures, mitochondria, nucleus, cytoplasm, cell membrane, and cell environment; and the average Raman spectra for the cell as a whole for human normal colon cells CCD-18Co, human cancer colon cells CaCo-2, and human cancer colon cells CaCo-2 upon supplementation with simvastatin, lovastatin, and mevastatin in 10 μM concentration for 24 h.

**Figure 10:**
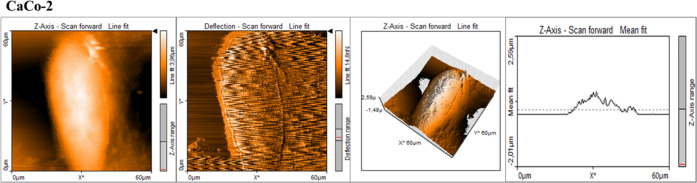
AFM topography maps of CaCo-2 with deflection maps, 3D topography, and curves related to the topography measurements for forward and backward traces. The scale bar is equal to 60 μm and is the same for all cell images, topography 3D, and deflection maps.

**Figure 11:**
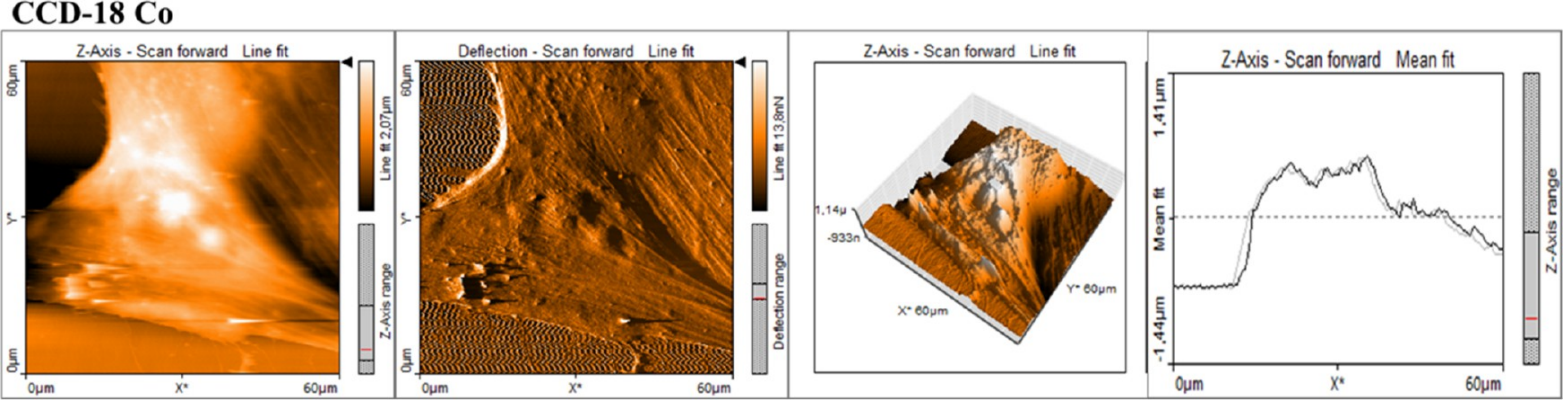
AFM topography maps of CCD-18Co with deflection maps, 3D topography, and curves related to the topography measurements for forward and backward traces. The scale bar is equal to 60 μm and is the same for all cell images, topography 3D, and deflection maps.

**Figure 12:**
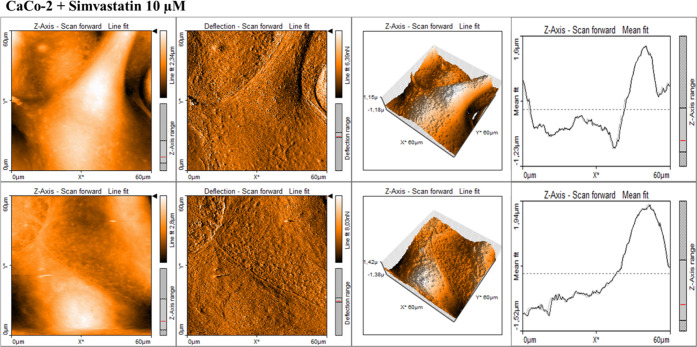
AFM topography maps of CaCo-2 supplemented by simvistatin (10 μM) for 24 h (upper panel) and 48 h (lower panel) with deflection maps, 3D topography, and curves related to the topography measurements for forward and backward traces. The scale bar is equal to 60 μm and is the same for all cell images, topography 3D, and deflection maps.

**Figure 13:**
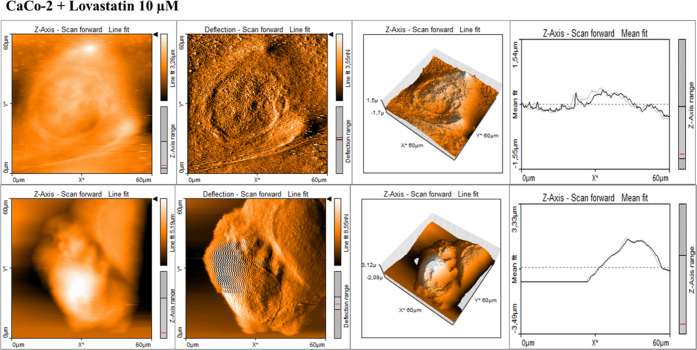
AFM topography maps of CaCo-2 supplemented by lovastatin (10 μM) for 24 h (upper panel) and 48 h (lower panel) with deflection maps, 3D topography, and curves related to the topography measurements for forward and backward traces. The scale bar is equal to 60 μm and is the same for all cell images, topography 3D, and deflection maps.

**Figure 14:**
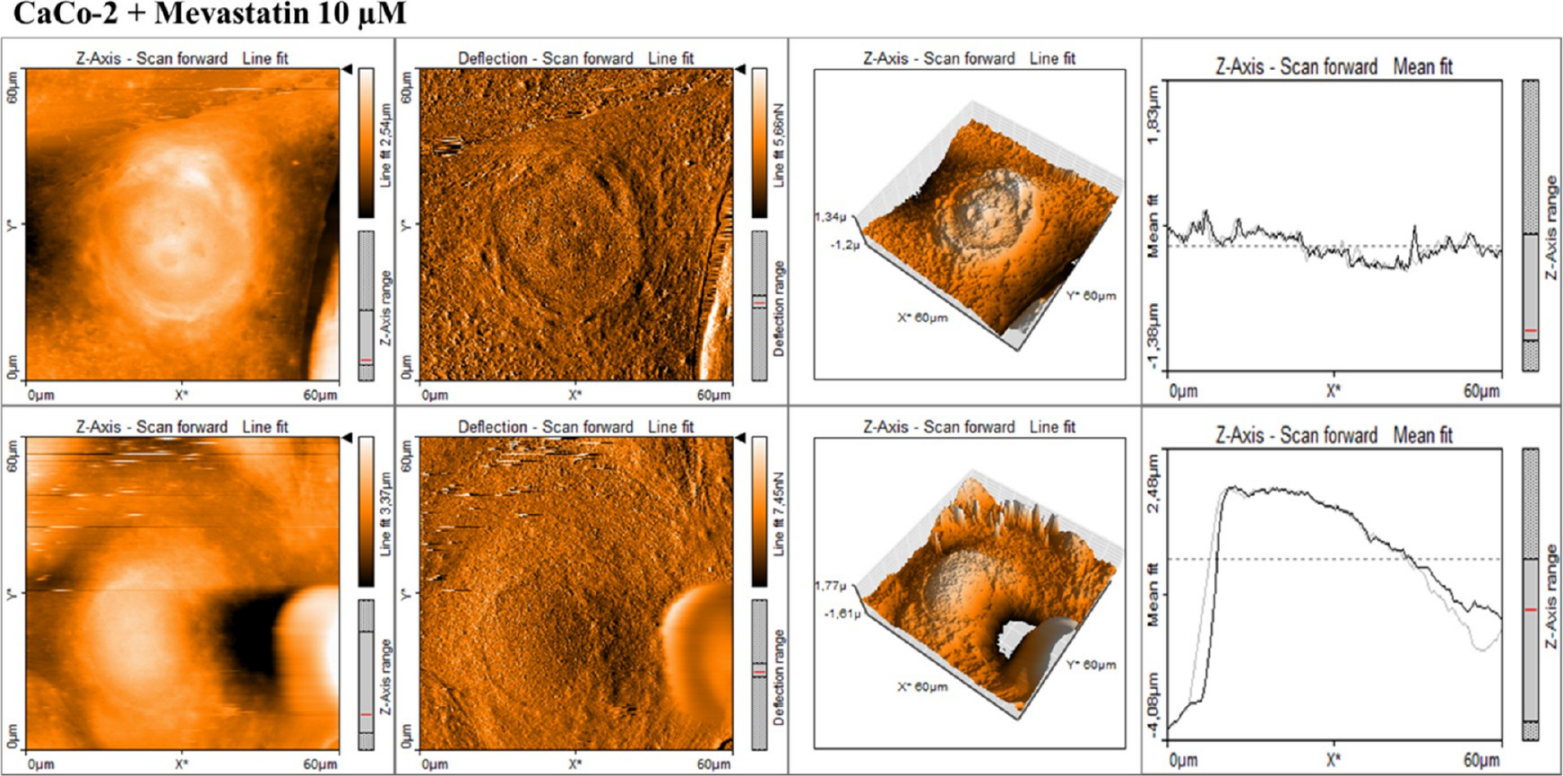
AFM topography maps of CaCo-2 supplemented by mevastatin (10 μM) for 24 h (upper panel) and 48 h (lower panel) with deflection maps, 3D topography, and curves related to the topography measurements for forward and backward traces. The scale bar is equal to 60 μm and is the same for all cell images, topography 3D, and deflection maps.

Investigations regarding cell biochemistry were extended with analysis of nanomechanical properties of human colon cells: normal, cancer, and cancer supplemented by statins. Figures 10–14 present the data obtained during AFM measurements: topography maps, deflection maps, topography maps in the 3D visualization mode, and data for forward and backward trace measurements.

## Discussion

To be able to perform clinical trials, a series of tests should be carried out to determine the activity of cells in terms of their metabolism and proliferation after exposure to specific substances. This is necessary because on this basis it is possible to determine whether a given chemical is producing a cytotoxic response. Initially, viability tests were developed to incorporate compounds such as 5-bromo-2-deoxyuridine (BrdU) or [H]-thymidine into the structure of DNA. Due to the inconvenience of this type of test related to the need to use radioactive materials, expensive equipment, or a time-consuming procedure, colorimetric methods have been developed. The basis of this method is the phenomenon observed for tetrazolium salts, which can be transformed by living cells as electron acceptors. As a result of this transformation, colored formazan compounds are formed. The first salt to be used in the colorimetric tests is 3-[4,5-dimethylthiazol-2-yl]-2,5-diphenyltetrazolium bromide, known as the MTT salt. It is a positively charged compound, thanks to which it easily penetrates the cell, where it is reduced to a water-insoluble formazan compound. However, this method is also not perfect due to the need to dissolve the formazan compound crystals in an organic solvent. For this reason, a method was developed in which the MTT salt was replaced with the 2,3-bis-[2-methoxy-4-nitro-5-sulfophenyl]-2*H*-tetrazoli-5-carboxanilide sodium salt, more widely known as the XTT salt. Unlike MTT, the XTT salt, when it enters the cell, is transformed into a product that can be dissolved in an aqueous medium. XTT, unlike the MTT salt, has a negative charge, so its permeability to the cell interior is low. This results in a reduction either at the cell surface or in the plasma membrane by the transmembrane electron transport chain. In Figure 4, we have presented the results of XTT comparison of the percent viability for Caco-2 human colon cancer cells supplemented with different concentrations of lovastatin in two different time intervals with the standard deviation ±SD, which allow us to determine the concentration of statin used in our experiments.

Figures 5–9 show the Raman imaging and Raman spectroscopy analysis of human colon single cells. One can see from Figures 5–9 that based on the Raman spectra for each measurement the main biochemical components of single human colon cells can be identified. The fingerprint region of Raman spectra provides complex information on the biochemical composition of the analyzed sample, e.g., the peak at 755 cm^–1^ is associated with nucleic acids, DNA, tryptophan, and nucleoproteins;^71^ the peak at ca. 850 cm^–1^ can be assigned to tyrosine;^47,71^ the sharp peak at 1004 cm^–1^ corresponds to the aromatic amino acid phenylalanine;^48,72−76^ the peak at 1126 cm^–1^ is typical for saturated fatty acids and cytochrome *c*; the band at 1304 cm^–1^ corresponds to deformation vibration of lipids, adenine, and cytosine;^48,72−76^ the band at 1444/1452 cm^–1^ is typical for lipids and proteins; and the peak at 1585 cm^–1^ is typical for CN_2_ scissoring and NH_2_ rock vibrations of mitochondria and phosphorylated proteins.^48,72−76^ In the Raman spectra, the peaks typical for proteins also can be observed in a form of the amide I (C=O stretch) near 1656 cm^–1^, amide II (N–H bend + C–N stretch) near 1557 cm^–1^, and very weak amide III bands (C–N stretch + N–H bend) near 1260 cm^–1^.^48,72−76^ The high-frequency peaks originate in the symmetric and antisymmetric stretching vibrations of C–H bonds found in lipids, glycogen, proteins, RNA, and DNA. Lipids and fatty acids, including the unsaturated fraction, can be seen at 2845, 2880, and 3009 cm^–1^. Protein contribution, in the high-frequency region, is observed at 2875, 2888, 2919, and 2926 cm^–1^.

Based on the Raman data obtained for normal cells, cancer cells, and cancer cells supplemented by statin, we can compare the vibrational features of human colon cells using the average spectra calculated for cells as a whole and the Raman band intensity ratios calculated for the main building blocks of biological samples: proteins, nucleic acids, and lipids.

**Figure 15:**
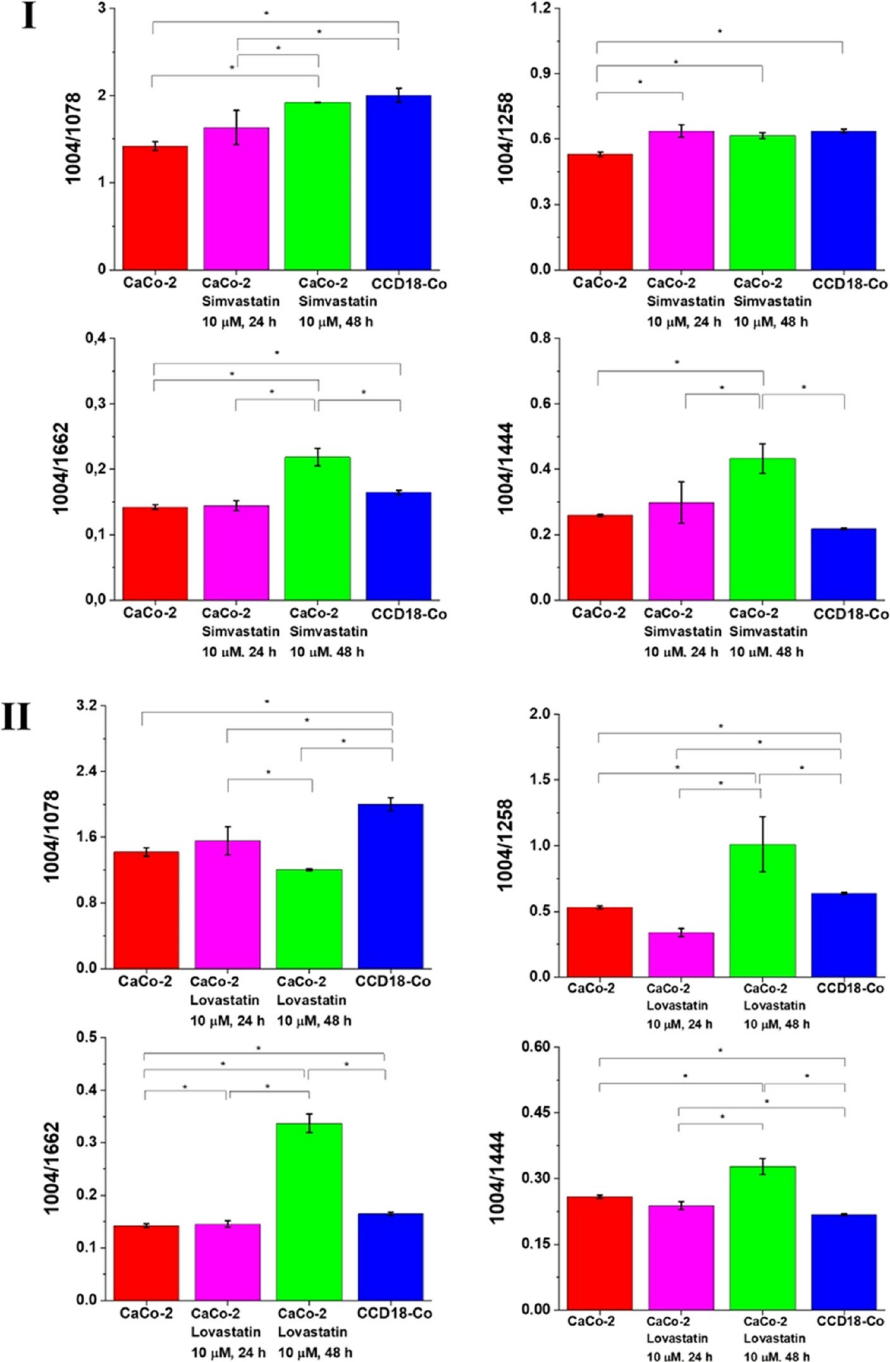

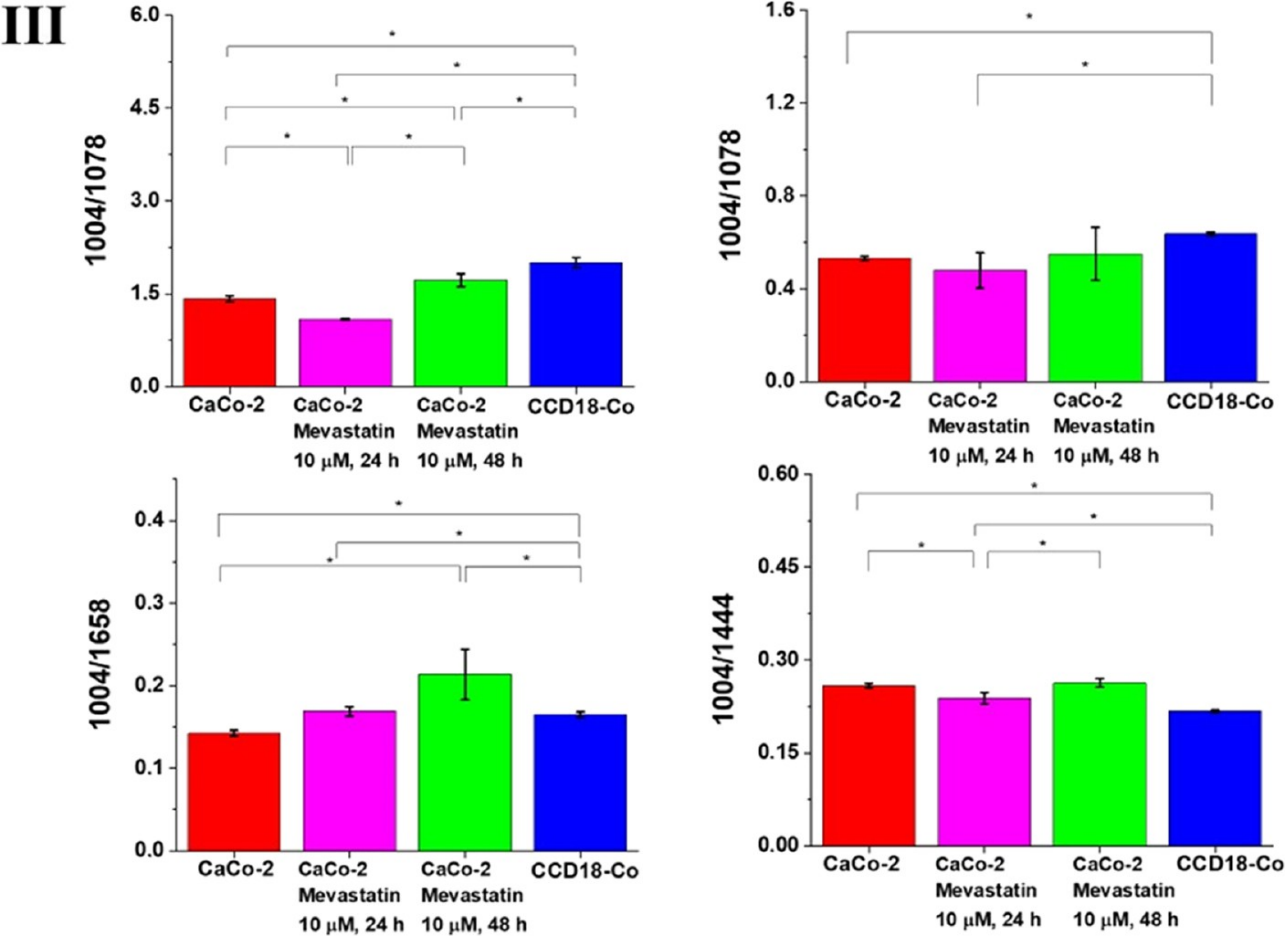
Raman band intensity ratios for selected Raman bands corresponding to nucleic acids 1004/1078, proteins 1004/1257 and 1004/1658, and proteins and lipids 1004/1444 for four groups of human colon cells: normal human colon cells CCD-18-Co: control group (labeled CCD-18Co, blue), cancer human colon cells CaCo-2 (labeled CaCo-2, red), cancer human colon cells CaCo-2 incubated with statins at the concentration of 10 μM for 24 h (labeled CaCo-2 simvastatin (I), lovastatin (II), or mevastatin (III), 10 μM, 24 h, magenta), and cancer human colon cells CaCo-2 incubated with statins at the concentration of 10 μM for 48 h (labeled CaCo-2 simvastatin (I), lovastatin (II), or mevastatin (III), 10 μM, 48 h, green); statistically significant data are marked with an asterisk (*). The Raman ratios have been calculated based on the data for six cells from each variant.

Figure 15 shows the Raman band intensity ratios for selected Raman bands corresponding to nucleic acids 1004/1078, proteins 1004/1257 and 1004/1658, and proteins and lipids 1004/1444 for four groups of human colon cells: normal human colon cells CCD-18-Co: control group (labeled CCD-18Co, blue), cancer human colon cells CaCo-2 (labeled CaCo-2, red), cancer human colon cells CaCo-2 incubated with statins at the concentration of 10 μM for 24 h (labeled CaCo-2 simvastatin, lovastatin, or mevastatin, 10 μM, 24 h, magenta), and cancer human colon cells CaCo-2 incubated with statins at the concentration of 10 μM for 48 h (labeled CaCo-2 simvastatin, lovastatin, or mevastatin, 10 μM, 48 h, green); statistically significant data were marked with an asterisk (*).

One can see from Figure 15 that the biochemical composition of normal cells, cancer cells, and cancer cells supplemented with statins is different and the differences are observed for all of the main chemical substituents: nucleic acids, proteins, and lipids.

In the first step of the analysis of the differences between normal and cancer cells, it must be underlined that one of the factors responsible for the induction of cancer transformation comprise reactive oxygen species (ROS). The imbalance between the production of ROS and the efficiency of antioxidant systems leads to oxidative stress and, consequently, damage of DNA, proteins, and lipids. Each day, the content of the human colon can be described as a diverse mix of bile, mucus, gut microflora, fermentation products, unabsorbed food, and products of metabolism, including toxins, mutagens, and dissolved gases. In such an environment, the colon mucosa is constantly exposed to dietary oxidants and a variety of bacteria. Permanent exposure of the mucosa and the organism itself to unfavorable conditions may lead to uncontrolled oxidative stress and DNA damage, which consequently may lead to the development of cancer disease.

Under homeostasis conditions, ROS act as mediators and regulators of metabolism—they induce cell differentiation, activate many genes, including oncogenes, induce apoptosis, influencing the synthesis, release, or inactivation of endothelial vasodilator factor (EDRF), have a dilating effect or contracting the wall of blood vessels, increase the permeability of capillary walls, and stimulate the transport of glucose to cells and serotonin to platelets. They influence the transmission of signals to cells and inside cells. They can become secondary transmitters in the process of both cell growth and death. They activate proteins that direct cell division (mitogenic activated protein). They take part in the body’s defense processes. Peroxides also regulate the synthesis of prostanoids.

Excessive production of ROS and depletion by the body’s antioxidant reserves is a phenomenon called “oxidative stress”. Oxidative stress leads to protein oxidation, which modifies their amount and structure and disrupts their function in the human body. Other main human body building components can also be damaged by ROS. The oxidation of lipids, damage to nucleic acids, depolymerization of hyaluronic acid, and the accumulation of IgG can be observed. ROS also inactivate protease inhibitors, which increases the proteolytic effect of these tissue enzymes.

As was mentioned above, the high concentrations of ROS trigger chain reactions, intensifying the processes of damaging biomolecules. Under ROS conditions, e.g., the residues of polyunsaturated acids undergo oxidation and fatty acids, which are part of the phospholipids, change cell membrane properties. Products of nonenzymatic peroxidation of lipids change the physical properties of cell membranes, which can lead to their damage.

Moreover, at the molecular level, ROS cause collagen degradation, disorders of the synthesis and inactivation of proteoglycans, enzyme inactivation, DNA strand breaks, formation of guide mutations to cancer changes, inhibition of oxidative phosphorylation in mitochondria, structure disorder cytoskeleton (actin polymerization, disruption of microfiber laments), modification of antigenic property of cells, and disturbance of intracellular calcium homeostasis.

Based on the data obtained using Raman spectroscopy and imaging for proteins, one can see in Figure 15 that the amount of this class of compounds was different for normal and cancer human colon cells and was modulated by the adding of statins. In the presented analysis, the intensity of the peak 1004 cm^–1^ was kept constant, which means that the decrease in each ratio correlates with the increase in the amount of the main building compounds of human colon cells: nucleic acids, proteins, and lipids.

In Figure 15, for CaCo-2 cancer cells, one can observe the lower intensities for ratios 1004/1257 and 1004/1658 compared to CCD-18Co cells. Such results were expected taking into account the fact that the development of cancer is associated with the overexpression of proteins. However, for cancer cells incubated with statins in 10 μM concentration, we noticed the statistically significant increase of analyzed ratios. This finding suggests that statin-induced inhibition of protein synthesis and the same protein-dependent mechanism for cell death should be underlined.^77^ Protein synthesis is one of the most complicated biochemical processes undertaken by the cell, requiring approximately 150 different polypeptides and 70 different RNAs. In addition, protein synthesis can be stopped when only a small fraction of the ribosome is inactivated by certain ribotoxins or when kinases associated with oxidative stress are activated.^78^ The comparison between untreated human colon cells and cancer human colon cells upon statin supplementation shows that adding of statins effectively decreases the cell’s protein level (the ratios 1004/1258 and 1004/1658 increase), especially for a longer incubation time of 48 h. One can see from Figure 15 that the strongest effect was observed for simvastatin.

Based on the data presented in Figure 15 for bands typical for nucleic acids, one can notice that the intensity of the ratio typical for these compounds 1004/1078 decreases for CaCo-2 human cancer colon cells compared to the control group—CCD-18Co corresponding to the normal human colon cells. This finding confirms that the synthesis of nucleic acids in cancer cells is enhanced, which is the expected result. Moreover, analyzing Figure 15, one can notice that the adding of statins modulates the amount of nucleic acids observed in CaCo-2 cancer cells. The ratio 1004/1078 increases, confirming the reduction of the DNA/RNA amount. Moreover, the concentration and incubation time dependence was observed. This finding is supported by scientific literature confirming, using traditional molecular biology methods, that decreased levels of DNA for cells interacting with statins are typical.^79,80^ The strongest effect was observed for simvastatin.

The statistically significant differences between normal human colon cells, cancer human colon cells, and cancer human colon cells upon statin supplementation have been found also for lipid components of analyzed samples. It is known that statins modulate the lipid composition of cells and tissues due to the influence on the cholesterol level (in general, statins represent HMG-CoA reductase inhibitors and are widely used for the treatment of hypercholesterolemia) and the reduction of triglyceride concentrations. Results obtained based on the intensity of Raman peaks related to lipids (peak at ca. 1444 cm^–1^) confirmed that a decreasing intensity of peaks typical for lipids for cells treated by statins is observed, and this effect is time and dose dependent^81^ (see Figure 15). The strongest effect was observed for simvastatin.

**Figure 16:**
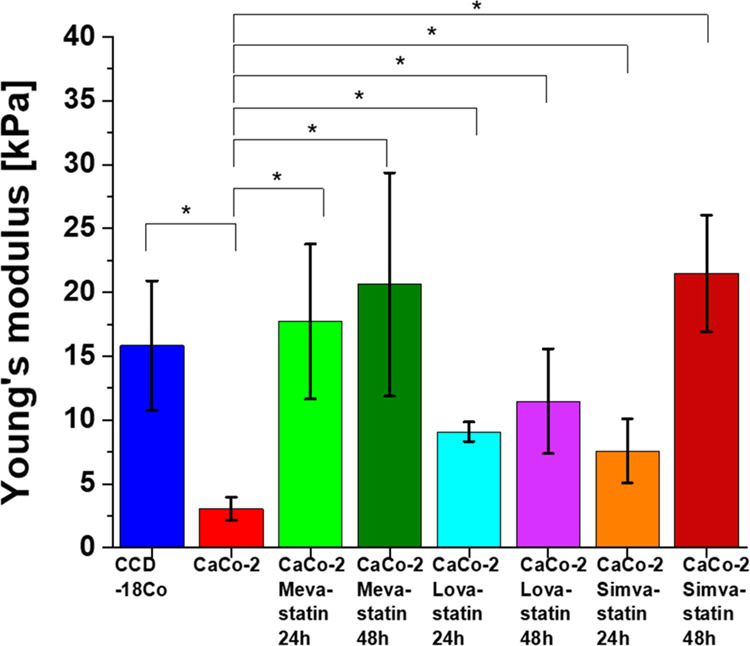
Young’s modulus values calculated for CCD-18Co (blue); CaCo-2 (red); CaCo-2 supplemented by mevastatin (10 μM, 24 h) (light green) and mevastatin (10 μM, 48 h) (dark green); lovastatin (10 μM, 24 h) (turquoise); lovastatin (10 μM, 48 h) (violet); simvastatin (10 μM, 24 h) (orange); and simvastatin (10 μM, 48 h) (brown). Young’s modulus values were calculated for the cell as a whole.

Figure 16 shows histograms related to Young’s modulus calculated for each type of analyzed sample.

One can see from Figure 16 that the cancer human colon cells CaCo-2 are more elastic compared to normal human colon cells CCD-18Co and that the adding of statins in 10 μM concentration modulates the nanomechanical properties of cancer cells. The supplementation using statins changed the elasticity of cancer cells, and Young’s modulus values are more comparable to the elasticity of normal CCD-18 human colon cells. Based on ANOVA test groups, CCD-18Co and CaCo-2 cells upon statin supplementation are not significantly different, while differences between CaCo-2 cells without and with statin supplementations are statistically significant. This finding confirms that changes in skeleton organization of analyzed cells upon statin supplementation occurred. The obtained results are consistent with literature data, which confirm the higher flexibility of cancer cells compared to normal ones.^82^ Quantitatively, the results in Figure 16 proved that the value of Young’s modulus for cancer cells is approximately 20% lower than for healthy cells. Supplementation with simvastatin causes a change in the value of Young’s modulus; for 24 h supplementation, there is a 2.5-fold increase in value, and for 48 h supplementation, there is a 7-fold increase in relation to cancer cells not subjected to supplementation. Supplementation with lovastatin also causes a change in the value of Young’s modulus; for 24 h supplementation, there is a 3-fold increase in value, and for 48 h supplementation, there is a 4-fold increase in relation to cancer cells not subjected to supplementation. Supplementation with mevastatin causes a change in the value of Young’s modulus; for 24 h supplementation, there is a 6-fold increase in value, and for 48 h supplementation, there is a 7-fold increase in relation to cancer cells not subjected to supplementation.

## Conclusions

The results proved that Raman imaging and spectroscopy are capable of differentiating human normal CCD-18Co and cancerous CaCo-2 colon cells and that vibrational spectra can be effectively used to efficiently and accurately classify single cells.

Based on the Raman spectra, we visualized the main substructures of single cells: nucleus, lipid structures, mitochondria, cytoplasm, and cell membrane.

Atomic force microscopy allowed us to characterize the nanomechanical properties of normal CCD-18Co and cancerous CaCo-2 human colon cells without and upon mevastatin supplementation.

The use of AFM to characterize elastic properties of normal and cancer cell lines justifies the idea of using nanomechanical parameters to track the changes typical for tumor development and antitumor treatment.

*In vitro* studies have shown that statins inhibit tumor growth and induce apoptosis in colon cancer cell lines.

Accumulating evidence suggests that the long-term use of lipophilic statins may also affect the overall incidence of cancer or the incidence of certain types of cancer. Moreover, statins may increase the sensitivity to chemotherapy and influence clinical outcomes in patients who have already been diagnosed with cancer.

The translation of the molecular information included in Raman spectra into an objective clinical diagnosis is the most important challenge for Raman spectroscopy in the future. Many optical imaging and microscopy techniques nowadays used in medical diagnostics (including the gold standard histopathology) can identify diseased cells based on their morphology or even interaction with specific stains or antibodies. However, they are based on subjective interpretation and thus are prone to high interobserver variability. The presented study proved that Raman spectroscopy can measure both morphological and chemical information of samples to provide objective diagnosis of samples obtained from cancer-diagnosed patients.

The relatively low signal of Raman spectroscopy and biological samples’ autofluorescence have been main weaknesses for clinical translation. However, recent advances in multimodal imaging demonstrated the ability to obtain high-contrast molecular images suitable for objective diagnosis for samples with clinically relevant dimensions and at speeds compatible with clinical use.
